# Psychometric Properties of The Fertility Quality of Life Instrument in Infertile Iranian Women

**DOI:** 10.22074/ijfs.2016.4696

**Published:** 2016-11-01

**Authors:** Saman Maroufizadeh, Azadeh Ghaheri, Payam Amini, Reza Omani Samani

**Affiliations:** Department of Epidemiology and Reproductive Health, Reproductive Epidemiology Research Center, Royan Institute for Reproductive Biomedicine, ACECR, Tehran, Iran

**Keywords:** Infertility, Quality of Life, Validity, Reliability

## Abstract

**Background:**

Infertility and its treatment can have a considerable effect on a person’s quality of life (QoL). The Fertility QoL (FertiQoL) questionnaire is currently the most frequently used instrument to measure QoL in people with fertility problems. This study aims to examine the reliability and validity of the FertiQoL in infertile Iranian women.

**Materials and Methods:**

This cross-sectional study included 155 women with fertility problems in a referral fertility clinic in Tehran, Iran from January to March 2014. A battery of instruments was used: FertiQoL, Satisfaction with Life Scale (SWLS), Hospital Anxiety and Depression Scale (HADS), and a demographic questionnaire. Construct validity of the scale was evaluated using confirmatory factor analysis (CFA). We assessed internal consistency with Cronbach’s alpha and convergent validity was examined by correlating the FertiQoL with SWLS and HADS.

**Results:**

The results of the CFA generally supported the four-factor model of Core FertiQoL and two-factor model of Treatment FertiQoL. Both FertiQoL modules and their subscales revealed acceptable internal consistency that ranged from 0.643 to 0.911. However, the FertiQoL might be improved if Q15 and T2 items were removed from the scale. These items had low loadings on the Relational and Environment factors which decreased their internal consistency. The FertiQoL and their subscales significantly correlated with both SWLS and HADS, which confirmed convergent validity.

**Conclusion:**

The Persian version of the FertiQoL is a valid, reliable instrument to measure QoL in infertile women and seems to perform as well as the original English Version.

## Introduction

Infertility is a global public health issue that affects approximately 10-15% of reproductive-aged couples worldwide (1). It reduces quality of life (QoL), especially through negative psychosocial and cultural consequences. Often-cited repercussions of infertility are depression, anxiety, social isolation and deprivation, marital instability, loss of self-esteem and self-confidence, loss of gender identity, loss of control, and feeling of self-blame and guilt (2-4). Growing bodies of research have shown that infertility and its treatments have a significant negative impact on a person’s QoL (5-10). Due to this impact, assessing QoL in infertile patients, especially for women is important (7).

The World Health Organization (WHO) has defined QoL as ‘an individual's perception of their position in life in the context of the culture and value systems in which they live and in relation to their goals, expectations, standards and concerns’ (11). QoL can be assessed by both generic and disease-specific tools (12). Previously, various generic self-reported instruments have been used to assess QoL in infertile patients (13, 14). Recently, an international group of experts in several countries and from various professions developed the Fertility QoL (FertiQoL) tool, which is disease-specific and assesses QoL in men and women that suffer from infertility (15).

The FertiQoL tool consists of two modules: Core FertiQoL and Treatment FertiQoL. The Core FertiQoL module assesses the impact of infertility in diverse life areas such as general health, self-esteem, emotions, partnership, family and social relationships, work life, and future life plans. The optional Treatment FertiQoL module assesses the burden or tolerability of fertility treatment. The FertiQoL has been used in various cultures and populations, and has been translated into 26 languages. However, few studies examined the psychometric properties of the FertiQoL. Initial psychometric properties of the FertiQoL were evaluated by Boivin et al. (15) in the USA, Australia/New Zealand, Canada and the UK. Their study demonstrated acceptable validity and reliability. In another study, reliability and convergent validity of the Dutch version of the FertiQoL were evaluated in infertile women. The Dutch version of the FertiQoL showed satisfactory internal consistency and had a negative correlation with anxiety and depression, which indicated acceptable convergent validity (16). In the Portuguese population, the results of confirmatory factor analysis (CFA) showed a good fit to the original measurement model and all FertiQoL domains were reliable (Cronbach’s alpha: 0.72 to 0.90) (17).

To the best of our knowledge, no studies evaluated the psychometric properties of the Persian version of the FertiQoL. Therefore, the present study aimed to examine the reliability and validity of the FertiQoL in infertile Iranian women.

## Materials and Methods

### Patients

We conducted this cross-sectional study at Royan Institute, Tehran, Iran from January 2014 to March 2014. The Infertility Clinic of Royan Institute is a referral infertility center which provides comprehensive treatment, including assisted reproduction techniques (ART). The inclusion criteria for this study were as follows: i. Women aged 18-45 years; ii. Diagnosed with couple infertility; and iii. Ability to read and write in Persian. Participants were selected through convenient sampling from infertile women in the embryo transfer stage of ART cycles at Royan Institute. The sample size was calculated at 120 patients, considering that 5 patients were necessary for each item (subject-to-item ratio: 5:1). As a rule of thumb, a minimum sample size of 100 would be enough for a psychometric study (18). In total, 155 women agreed to participate and completely filled out the questionnaires.

### Ethical approval

The Ethics Committee of Royan Institute, Tehran, Iran approved the study protocol. All participants were fully informed about the study’s scope and purpose, and the confidentiality of the data. Eligible women were also assured that the data would be used only for the purpose of the study and acceptance or refusal to participate in the research had no influence on their current or future treatments. A verbal informed consent was obtained from all participants before data collection.

### Instruments

#### Fertility Quality of Life Tool

FertiQoL is a self-report instrument that assesses QoL in individuals with fertility problems (15). FertiQoL is composed of two modules: the Core FertiQoL and Treatment FertiQoL. The Core FertiQoL module consists of 26 items. Two items are general and 24 items specific to infertility that cover four subscales of the QoL (i.e., 6 items per subscale). The four subscales are as follows: Emotional, Mind-Body, Relational, and Social. The optional Treatment FertiQoL module is composed of ten items that assess the following two subscales: Environment (6 items), and Tolerability (4 items). The FertiQoL yields 6 subscales and 2 total scores with a range of 0-100, with higher scores indicative of better QoL. The FertiQoL is a free to use instrument and the Persian version of FertiQoL is available at: www.fertiqol.org. The translation from English to Persian was performed by professional translators from Cardiff University. This paper’s first author assisted in the translation process by checking the Cardiff researchers’ word usage against local word use.

#### Satisfaction with Life Scale

The Satisfaction with Life Scale (SWLS) is a short 5-item instrument designed to measure global cognitive judgments of satisfaction with one’s life. Each item was scored on a 7-point Likert scale that ranged from 1 (strongly disagree) to 7 (strongly agree). Scale scores range from 5-35, with higher scores indicative of greater life satisfaction (19). The Persian version of the SWLS had adequate psychometric properties in the Iranian populations (20). The Cronbach’s alpha coefficient for the SWLS was 0.872 in the present study.

#### Hospital Anxiety and Depression Scale

The Hospital Anxiety and Depression Scale (HADS) is a 14-item self-report inventory composed of two subscales: Anxiety (HADS-A) and Depression (HADS-D). Both subscales of HADS consist of 7 items with each item scored on a 4-point Likert scale that ranges from 0 to 3. Subscale scores range from 0-21, with higher scores indicating higher level of anxiety and depression, respectively. We have used the Persian version of HADS in the present study. This version has previously been shown to have satisfactory reliability and validity (21). The Cronbach’s alpha coefficient for HADS-A was 0.840 whereas for HADS-D, it was 0.733 in the present study.

### Statistical analysis

The factor structure of the Core FertiQoL and Treatment FertiQoL were examined by CFA. These models were tested using covariance matrices and the maximum likelihood estimation method. Goodness of fit of models were assessed using the chi-square (χ2), relative chi-square (χ2/df), the comparative fit index (CFI), the root mean square error of approximation (RMSEA), and the standardized root mean square residual (SRMR). The χ2 statistic is the classical measure for evaluating model fitness, but it is highly sensitive to sample size (22). Therefore we have used χ2/df as an alternative index to examine the model fit. A χ2/df ratio of less than 3 is considered indicative of a good fit (23). For other goodness of fit indices, acceptable thresholds are CFI>0.90, RMSEA<0.07 and SRMR<0.08 (24). We have used Cronbach’s alpha to measure the internal consistency of the FertiQoL. Values above 0.80 were considered excellent, 0.70-0.80 satisfactory, and 0.60-0.70 acceptable (25). Convergent validity of the FertiQoL was assessed by calculating its Pearson correlation coefficients with SWLS and HADS.

All data analyses were performed using SPSS version 16.0 (SPSS Inc., Chicago, IL, USA), except for the CFAs, which were performed using Lisrel 8.80 (Scientific Software International, Inc., Lincolnwood, IL, USA). All statistical tests were two-tailed and a P value of less than 0.05 was considered statistically significant.

## Results

### Participant characteristics

The demographic and fertility characteristics of the women are presented in Table 1. The mean age of women was 31.03 ± 5.89 years. Among all participants, 45.8% had male factor infertility, 43.2% had a university education, 40.6% had no previous treatments, and 82.6% had no history of abortion. The mean duration of infertility was 6.25 ± 4.36 and 78.7% of women had primary infertility.

**Table 1:** Demographic and fertility characteristics of the participants (n=155)


		Mean ± SD or n (%)

Age (Y)		31.03 ± 5.89
Duration of infertility (Y)		6.25 ± 4.36
Cause of infertility		
	Male factor	71 (45.8)
	Female factor	29 (18.7)
	Both	26 (16.8)
	Unexplained	29 (18.7)
Type of infertility		
	Primary	122 (78.7)
	Secondary	33 (21.3)
Education level		
	Primary	29 (18.7)
	Secondary	59 (38.1)
	University	67 (43.2)
Failure of previous treatment		
	0	63 (40.6)
	1	41 (26.5)
	2	20 (12.9)
	3	20 (12.9)
	≥4	11 (7.1)
History of abortion		
	No	128 (82.6)
	Yes	27 (17.4)

### Confirmatory factor analysis

We used the CFAs to determine the goodness of fit for the four-factor model of Core FertiQoL and two-factor model of Treatment FertiQoL. Although the χ2 value of the Core FertiQoL model was not satisfactory (χ2=410.80, df=252, P<0.001), the relative chi-square (χ2/df=1.63) was satisfactory. Examination of other goodness of fit indices indicated that the model provided an acceptable fit to the data, with CFI=0.96, RMSEA=0.064, and SRMR=0.067. All factor loadings were significant, except for Q15 (0.16), which ranged from 0.36 to 0.97 (Table 2).

**Table 2:** Confirmatory factor analysis (CFA) of the Core Fertility Quality of Life (FertiQoL) in infertile women


	Subscale-item	Factor loading (SE)

	Emotional	
Q4	Do you feel able to cope with your fertility problems?	0.36 (0.10)
Q7	Do your fertility problems cause feelings of jealousy and resentment?	0.68 (0.09)
Q8	Do you experience grief and/or feelings of loss about not being able to have a child (or more children)?	0.91 (0.09)
Q9	Do you fluctuate between hope and despair because of fertility problems?	0.82 (0.08)
Q16	Do you feel sad and depressed about your fertility problems?	0.93 (0.08)
Q23	Do your fertility problems make you angry?	0.92 (0.09)
	Mind/body	
Q1	Are your attention and concentration impaired by thoughts of infertility?	0.75 (0.09)
Q2	Do you think you cannot move ahead with other life goals and plans because of fertility problems?	0.76 (0.09)
Q3	Do you feel drained or worn out because of fertility problems?	0.97 (0.09)
Q12	Do your fertility problems interfere with your day-to-day work or obligations?	0.61 (0.09)
Q18	Are you bothered by fatigue because of fertility problems?	0.89 (0.09)
Q24	Do you feel pain and physical discomfort because of your fertility problems?	0.65 (0.08)
	Relational	
Q6	Are you satisfied with your sexual relationship even though you have fertility problems?	0.50 (0.08)
Q11	Are you and your partner affectionate with each other even though you have fertility problems?	0.54 (0.10)
Q15	Have fertility problems strengthened your commitment to your partner?	0.16 (0.11)
Q19	Have fertility problems had a negative impact on your relationship with your partner?	0.82 (0.08)
Q20	Do you find it difficult to talk to your partner about your feelings related to infertility?	0.52 (0.09)
Q21	Are you content with your relationship even though you have fertility problems?	0.57 (0.09)
	Social	
Q5	Are you satisfied with the support you receive from friends with regard to your fertility problems?	0.51 (0.08)
Q10	Are you socially isolated because of fertility problems?	0.94 (0.10)
Q13	Do you feel uncomfortable attending social situations like holidays and celebrations because of your fertility problems?	0.95 (0.09)
Q14	Do you feel your family can understand what you are going through?	0.35 (0.11)
Q17	Do your fertility problems make you inferior to people with children?	0.84 (0.10)
Q22	Do you feel social pressure on you to have (or have more) children?	0.82 (0.09)


SE; Standard error.

The CFA for two-factor model of Treatment FertiQoL showed a significant χ2 value (χ2=64.35, df=34, P=0.001). The relative chi-square was 1.89, which indicated that the model was an acceptable fit to data. The other fit indices were CFI=0.91, RMSEA=0.076, and SRMR=0.071. All factor loadings were significant, except for T2 (0.02), which ranged from 0.35 to 0.74 (Table 3).

### Reliability analysis

Table 4 shows Cronbach’s alpha coefficients of the Core FertiQoL, Treatment FertiQoL, and their subscales. Both module of FertiQoL and their subscales revealed acceptable internal consistency that ranged from 0.643 to 0.911.

### Convergent validity

In order to examine the convergent validity of the FertiQoL, we calculated Pearson correlation coefficients between FertiQoL, SWLS, and HADS (Table 5). As expected, the Core FertiQoL and their subscales showed significant positive correlation with the SWLS (range: 0.375 to 0.488) and negative correlation with the HADS-A (range:-0.488 to-0.632) and the HADS-A (range:-0.501 to-0.662), which indicated acceptable convergent validity.

**Table 3:** Confirmatory factor analysis (CFA) of the Treatment Fertility Quality of Life (FertiQoL) in infertile omen


	Subscale-item	Factor loading (SE)

	Environment	
T2	Are the fertility medical services you would like available to you?	0.02 (0.10)
T5	Do you feel the fertility staff understand what you are going through?	0.46 (0.08)
T7	Are you satisfied with the quality of services available to you to address your emotional needs?	0.65 (0.07)
T8	How would you rate the surgery and/or medical treatment(s) you have received?	0.57 (0.06)
T9	How would you rate the quality of information you received about medication, surgery and/or medical treatment?	0.60 (0.07)
T10	Are you satisfied with your interactions with fertility medical staff?	0.54 (0.08)
	Tolerability	
T1	Does infertility treatment negatively affect your mood?	0.66 (0.11)
T3	How complicated is dealing with the procedure and/ or administration of medication for your infertility treatment(s)?	0.35 (0.09)
T4	Are you bothered by the effect of treatment on your daily or work related activities?	0.73 (0.10)
T6	Are you bothered by the physical side effects of fertility medications and treatment?	0.74 (0.11)


SE; Standard error.

**Table 4:** Descriptive statistics and Cronbach’s alpha coefficients of the Core Fertility Quality of Life (FertiQoL) and Treatment FertiQoL in infertile women


		Reliability analysis		Descriptive statistics	
		
	Subscale	Number of items	Cronbach’s alpha	Mean	SD

**Core FertiQoL**	Emotional	6	0.817	53.4	21.4
	Mind/Body	6	0.821	62.1	21.4
	Relational	6	0.643	70.9	16.5
	Social	6	0.750	63.9	21.1
	Total scale	24	0.910	62.6	16.9
**Treatment FertiQoL**	Environment	6	0.672	61.3	14.2
	Tolerability	4	0.643	54.0	19.2
	Total scale	10	0.693	58.4	12.9


**Table 5:** Pearson correlation coefficients between FertiQoL and the SWLS, HADS-A, and HADS-D in infertile women (n=155)


			HADS	
			
	Subscale	SWLS	HADS-A	HADS-D

**Core FertiQoL**	Emotional	0.375^***^	-0.503^***^	-0.529^***^
	Mind/body	0.421^***^	-0.576^***^	-0.622^***^
	Relational	0.440^***^	-0.488^***^	-0.501^***^
	Social	0.410^***^	-0.550^***^	-0.562^***^
	Total scale	0.488^***^	-0.632^***^	-0.662^***^
**Treatment FertiQoL**	Environment	0.251^**^	-0.146	-0.157
	Tolerability	0.246^**^	-0.262^***^	-0.382^***^
	Total scale	0.313^***^	-0.253^**^	-0.332^***^


FertiQoL; Fertility Quality of Life, HADS; Hospital Anxiety Depression Scale, SWLS; Satisfaction with Life Scale, HADS-A; HADS-Anxiety, HADS-D; HADS-Depression, ** ; P<0.01, and *** ; P<0.001.

### Relationship of the FertiQoL with demographic characteristics

As presented in Table 6, there were no significant relationships between Core FertiQoL and age (P=0.620), durations of infertility (P=0.165), and history of abortion (P=0.927). A significant difference existed among the groups in terms of their treatment failures on the Core FertiQoL; Duncan’s post hoc test revealed that women with two failures in treatment had lower QoL than women without failure and women with ≥4 failures (P<0.05). There was a direct relationship between Core FertiQoL and educational level (P=0.009). Regarding the cause of infertility, the mean Core FertiQoL was lower among women who had both factors and unknown cause of infertility than other participants (P<0.05). The relationships between Treatment FertiQoL and demographic characteristics are shown in Table 6.

**Table 6:** Relationship of Fertility Quality of Life (FertiQoL) with demographic and clinical characteristics in infertile women


		Core FertiQoL					Treatment FertiQoL		
		Emotional	Mind/Body	Relational	Social	Total	Environment	Tolerability	Total

Age (Y)									
	<30	49.6 ± 18.7	60.8 ± 19.2	72.3 ± 14.7	62.6 ± 18.8	61.3 ± 14.2	61.9 ± 13.1	54.1 ± 17.3	58.8 ± 11.8
	30-35	55.6 ± 22.5	60.7 ± 24.5	72.4 ± 15.9	64.0 ± 25.3	63.2 ± 19.7	61.2 ± 13.1	55.2 ± 20.7	58.8 ± 12.7
	≥35	58.9 ± 24.2	66.6 ± 22.1	66.2 ± 19.9	66.4 ± 20.9	64.5 ± 19.0	60.2 ± 17.7	52.4 ± 21.5	57.1 ± 15.3
P value		0.070	0.356	0.146	0.664	0.620	0.854	0.809	0.790
Duration infertility (Y)									
	<3	56.5 ± 17.9	66.4 ± 19.0	72.2 ± 17.4	69.2 ± 20.4	66.1 ± 15.8	60.1 ± 11.6	52.8 ± 17.9	57.2 ± 10.8
	3-6	53.3 ± 22.8	61.7 ± 21.4	71.9 ± 17.9	64.4 ± 20.5	62.8 ± 17.3	57.9 ± 15.1	51.8 ± 19.8	55.5 ± 13.8
	≥6	51.0 ± 22.3	59.2 ± 23.0	68.6 ± 14.0	59.1 ± 21.6	59.5 ± 17.1	66.0 ± 14.1	57.4 ± 19.4	62.6 ± 12.4
P value		0.459	0.266	0.480	0.064	0.165	0.008	0.266	0.010
Cause of infertility									
	Male factor	55.8 ± 21.4	65.7 ± 20.4	72.5 ± 16.4	65.2 ± 20.4	64.8 ± 16.7	62.6 ± 14.1	58.0 ± 18.5	60.7 ± 13.0
	Female factor	59.6 ± 22.2	67.0 ± 24.2	75.3 ± 15.8	70.7 ± 21.9	68.1 ± 17.8	64.5 ± 14.8	53.0 ± 21.0	59.9 ± 13.6
	Both	47.9 ± 20.6	53.2 ± 19.7	66.5 ± 18.3	62.5 ± 20.7	57.5 ± 16.4	61.4 ± 12.5	49.3 ± 19.6	56.5 ± 12.1
	Unexplained	46.3 ± 19.1	56.5 ± 19.7	66.2 ± 14.4	55.2 ± 20.2	56.0 ± 14.3	54.9 ± 14.1	49.4 ± 17.2	52.7 ± 11.1
P value		0.038	0.017	0.074	0.037	0.010	0.046	0.090	0.028
Type of infertility									
	Primary	52.8 ± 21.2	62.0 ± 21.5	71.2 ± 17.0	63.5 ± 22.1	62.4 ± 17.3	62.7 ± 13.4	54.9 ± 19.4	59.6 ± 11.9
	Secondary	55.7 ± 22.1	62.8 ± 21.6	69.4 ± 14.5	65.4 ± 16.9	63.3 ± 15.4	56.1 ± 16.2	50.8 ± 18.3	53.9 ± 15.3
P value		0.494	0.850	0.580	0.645	0.776	0.017	0.277	0.026
Educational level									
	Primary	48.7 ± 21.0	58.8 ± 22.8	66.2 ± 13.6	58.0 ± 22.6	57.9 ± 16.6	66.7 ± 12.1	56.0 ± 20.9	62.4 ± 12.5
	Secondary	50.1 ± 21.7	58.1 ± 21.9	68.6 ± 16.5	61.2 ± 20.7	59.5 ± 16.5	60.0 ± 14.5	53.5 ± 21.1	57.4 ± 14.5
	University	58.4 ± 20.5	67.2 ± 19.6	74.8 ± 17.0	68.8 ± 20.0	67.3 ± 16.4	60.1 ± 14.5	53.5 ± 16.8	57.5 ± 11.4
P value		0.038	0.037	0.027	0.033	0.009	0.078	0.819	0.173
Failure of treatment									
	0	59.7 ± 17.9	68.8 ± 19.7	70.4 ± 15.3	67.9 ± 19.4	66.7 ± 14.6	63.7 ± 13.3	60.2 ± 17.0	62.3 ± 11.9
	1	52.0 ± 21.3	60.2 ± 22.0	70.5 ± 18.9	61.7 ± 23.2	61.1 ± 18.5	58.5 ± 14.3	45.6 ± 18.9	53.4 ± 12.0
	2	39.8 ± 23.6	51.5 ± 21.0	67.1 ± 18.6	56.3 ± 23.9	53.6 ± 18.0	60.0 ± 17.5	47.5 ± 18.4	55.0 ± 12.3
	3	45.2 ± 19.9	55.6 ± 18.3	72.8 ± 13.2	61.7 ± 16.2	58.8 ± 14.4	60.8 ± 13.9	53.4 ± 15.2	57.9 ± 11.7
	≥4	62.1 ± 23.8	62.1 ± 25.2	78.0 ± 15.5	67.0 ± 22.8	67.3 ± 19.1	61.0 ± 13.7	62.5 ± 25.9	61.6 ± 17.9
P value		0.001	0.008	0.493	0.211	0.019	0.480	0.001	0.006
History of abortion									
	No	53.1 ± 22.0	62.3 ± 22.2	70.9 ± 17.0	63.9 ± 22.0	62.5 ± 17.5	62.8 ± 13.5	54.9 ± 19.5	59.6 ± 12.2
	Yes	55.1 ± 18.5	61.4 ± 17.9	70.8 ± 14.0	64.0 ± 16.6	62.8 ± 13.9	54.2 ± 15.5	49.8 ± 17.5	52.4 ± 14.7
P value		0.656	0.852	0.993	0.969	0.927	0.004	0.209	0.008


Values are mean ± SD.

## Discussion

The present study has aimed to evaluate the psychometrics properties of the FertiQoL in a sample of infertile women. FertiQoL is an infertility-specific questionnaire. In contrast to similar generic measures, it limits the factors that affect QoL to only infertility and no other stressful events. To our knowledge, this is the first study that has evaluated the factor structure of FertiQoL after a study by Melo et al. (17). The four-factor model of Core FertiQoL and two-factor model of Treatment FertiQoL were tested. In general, the Core and Treatment FertiQoL provided an acceptable fit to data. All factor loadings were significant, except for Q15 and T2. The model fit indices were acceptable similar to a study conducted by Melo et al. (17). The Core FertiQoL and their subscales showed satisfactory internal consistency, except for the Relational subscale (0.643) which had better reliability after removal of Q15 (0.689). The Treatment FertiQoL and their subscales showed acceptable internal consistency (0.6-0.7); at the same time reliability of Environment subscale improved after we removed item T2 (0.771). These findings indicated that some modifications for item Q15 and T2 might be needed in the scale to yield better internal consistency. A cross-cultural difference might contribute to these results.

Our finding confirmed the expected direct relationship between Core FertiQoL and SWLS, which indicated an acceptable convergent validity. As anticipated, the Core FertiQoL and its subscales negatively correlated with anxiety and depression. Infertile women with a high Core FertiQoL score reported lower levels of anxiety or depression and vice versa. These results supported previous studies and confirmed the convergent validity of Core FertiQoL (16, 26).

We also investigated the relationship between demographic characteristics and QoL. Although the difference was not statistically significant, on average, older women reported higher Core FertiQoL, Mind-Body, Emotional and Social subscales. Conversely, older women have reported lower Relational scores than younger women, but this difference was not significant. In general therefore, as women with infertility over 35 are considered old to be pregnant, their sexual relationship seems more pointless. This finding was roughly consistent with Aarts et al. (16). The results of this study did not show a significant relationship between Core FertiQoL and duration of infertility. The same results were reported by Rashidi et al. (13) and Keramat et al. (27). In contrast, women with lower infertility duration had lower Treatment FertiQoL. This result might be explained by the fact that infertile women become more aware of the treatment process over time.

A direct relationship was found with the Core FertiQoL and its subscales in terms of education level; in other words, the higher the education level, the greater the QoL. This result agreed with previous findings from Chachamovich et al. (7) and Rashidi et al. (13). Conversely, we observed lower Treatment FertiQoL among women with higher education. This result was inconsistent with the findings of Karabulut et al. (28). Women with two failures scored lower than other women on both Core and Treatment modules and their subscales, except for the Relational and Environment subscale. This results indicated that women with two failures might suffer from lower QoL and need to be supported by family, friends, and society (29). Psychological intervention, especially those that emphasize stress management and coping-skills training, might improve QoL in these women through affecting bio-psychological dimensions. We have found worse QoL in women whose source of infertility was both and unknown. Possibly when the problem is attributable to both there is no hope for gamete donation anymore. When the cause of infertility is unknown the roles are vague so the supportive role cannot be played by either of the couples to improve their QoL. Our study has found no association between Core FertiQoL and history of abortion. In contrast, women with abortion reported lower Treatment FertiQoL score than women with no abortion. This result may be explained by the fact that centers explain neither details of procedures nor the success rate of each procedure to the patients properly; this fact is what women with abortions know better. On the other hand these women are less assured about successful deliveries and expect the centers follow them until delivery rather than just releasing them when they are diagnosed pregnant.

Limitations of this study should be considered. First, the FertiQoL can separately assess the QoL in both women and men. Due to practical reasons, only the infertile women included in the study and their partners did not participate. We have only included women who were undergoing *in vitro* fertilization (IVF) treatment in the study. Those in the pre-treatment, diagnostic phase, or other ART were not investigated. Hence, generalization of the results might be affected by the sample. Second, this was a cross-sectional study and the causal relationship between QoL, SWLS, anxiety, depression, and infertility could not be established. Third, we did not examine test-retest reliability in this study.

## Conclusion

The Persian version of FertiQoL is a valid, reliable instrument for measuring QoL in infertile women that provide an exhaustive and comprehensive assessment of QoL related to fertility problems across diverse life areas. However, further psychometric studies are needed in diverse populations, especially in infertile men, including test-retest reliability.

## References

[1] Boivin J, Bunting L, Collins JA, Nygren KG (2007). International estimates of infertility prevalence and treatment-seeking: potential need and demand for infertility medical care. Hum Reprod.

[2] Cousineau TM, Domar AD (2007). Psychological impact of infertility. Best Pract Res Clin Obstet Gynaecol.

[3] van Balen F, Bos HM (2009). The social and cultural consequences of being childless in poor-resource areas. Facts Views Vis Obgyn.

[4] Maroufizadeh S, Karimi E, Vesali S, Omani Samani R (2015). Anxiety and depression after failure of assisted reproductive treatment among patients experiencing infertility. Int J Gynaecol Obstet.

[5] Verhaak CM, Smeenk JM, Evers AW, Kremer JA, Kraaimaat FW, Braat DD (2007). Women’s emotional adjustment to IVF: a systematic review of 25 years of research. Hum Reprod Update.

[6] Dancet EA, Nelen WL, Sermeus W, De Leeuw L, Kremer JA, D'Hooghe TM (2010). The patients' perspective on fertility care: a systematic review. Hum Reprod Update.

[7] Chachamovich JR, Chachamovich E, Ezer H, Fleck MP, Knauth D, Passos EP (2010). Investigating quality of life and health-related quality of life in infertility: a systematic review. J Psychosom Obstet Gynaecol.

[8] Schmidt L (2006). Psychosocial burden of infertility and assisted reproduction. Lancet.

[9] Ghaheri A, Shojaei Shahrokhabadi M, Zayeri F, Maroufizadeh S, Karimi M (2016). Relationship among life satisfaction, anxiety and fertility quality of life in women. Koomesh.

[10] Maroufizadeh S, Ghaheri A, Omani Samani R (2016). Factors associated with poor quality of life among Iranian infertile women undergoing IVF. Psychol Health Med.

[11] (1995). The World Health Organization quality of life assessment (WHOQOL): position paper from the World Health Organization. Soc Sci Med.

[12] Luckett T, King M, Butow P, Friedlander M, Paris T (2010). Assessing health-related quality of life in gynecologic oncology: a systematic review of questionnaires and their ability to detect clinically important differences and change. Int J Gynecol Cancer.

[13] Rashidi B, Montazeri A, Ramezanzadeh F, Shariat M, Abedinia N, Ashrafi M (2008). Health-related quality of life in infertile couples receiving IVF or ICSI treatment. BMC Health Serv Res.

[14] Chachamovich JL, Chachamovich E, Ezer H, Cordova FP, Fleck MM, Knauth DR (2010). Psychological distress as predictor of quality of life in men experiencing infertility: a cross-sectional survey. Reprod Health.

[15] Boivin J, Takefman J, Braverman A (2011). The fertility quality of life (FertiQoL) tool: development and general psychometric properties. Fertil Steril.

[16] Aarts JW, van Empel IW, Boivin J, Nelen WL, Kremer JA, Verhaak CM (2011). Relationship between quality of life and distress in infertility: a validation study of the Dutch FertiQoL. Hum Reprod.

[17] Melo C, Gameiro S, Canavarro M, Boivin J (2012). Does the FertiQoL assess quality of life?. Results from the validation of the Portuguese version of the FertiQoL. Hum Reprod.

[18] Tabachnick BG, Fidell LS (2007). Using multivariate statistics.

[19] Diener E, Emmons RA, Larsen RJ, Griffin S (1985). The satisfaction with life scale. J Pers Assess.

[20] Maroufizadeh S, Ghaheri A, Omani Samani R, Ezabadi Z (2016). Psychometric properties of the satisfaction with life scale (SWLS) in Iranian infertile women. Int J Reprod Biomed (Yazd).

[21] Montazeri A, Vahdaninia M, Ebrahimi M, Jarvandi S (2003). The Hospital Anxiety and Depression Scale (HADS): translation and validation study of the Iranian version. Health Qual Life Outcomes.

[22] Hu Lt, Bentler PM (1999). Cutoff criteria for fit indexes in covariance structure analysis: Conventional criteria versus new alternatives. Struct Equ Modeling.

[23] Kline RB (2011). Principles and practice of structural equation modeling.

[24] Steiger JH (2007). Understanding the limitations of global fit assessment in structural equation modeling. Pers Individ Dif.

[25] Nunnally JC, Bernstein IH (1994). Psychometric Theory.

[26] Kahyaoglu Sut H, Balkanli Kaplan P (2014). Quality of life in women with infertility via the FertiQoL and the Hospital Anxiety and Depression Scales. Nurs Health Sci.

[27] Keramat A, Masoomi SZ, Mousavi SA, Poorolajal J, Shobeiri F, Hazavhei SM (2013). Quality of life and its related factors in infertile couples. J Res Health Sci.

[28] Karabulut A, Özkan S, Oğuz N (2013). Predictors of fertility quality of life (FertiQoL) in infertile women: analysis of confounding factors. Eur J Obstet Gynecol Reprod Biol.

[29] Martins MV, Peterson BD, Almeida VM, Costa ME (2011). Direct and indirect effects of perceived social support on women's infertility-related stress. Hum Reprod.

